# Feasibility of patch-type wireless 12-lead electrocardiogram in laypersons

**DOI:** 10.1038/s41598-023-31309-0

**Published:** 2023-03-10

**Authors:** Sunyoung Yoon, Taerim Kim, Eunjin Kang, Sejin Heo, Hansol Chang, Yeoni Seo, Won Chul Cha

**Affiliations:** 1 Department of Digital Health, Samsung Advanced Institute for Health Science & Technology (SAIHST), Sungkyunkwan University, 115 Irwon-ro Gangnam-gu, Seoul, 06355 Republic of Korea; 2 Department of Emergency Medicine Samsung Medical Center, Sungkyunkwan University School of Medicine, 115 Irwon-ro Gangnam-gu, Seoul, 06355 Republic of Korea; 3 Department of Emergency Medicine Cheju Halla General Hospital, 65, Doryeong-ro63127, Jeju-si, Jeju-do Republic of Korea; 4 Department of International Health and Health Policy, Clinical & Public Health Convergence, Ewha Womans University, 52, Ewhayeodae-gil, Seodaemun-gu, Seoul, 03760 Republic of Korea; 5 Digital Innovation, Samsung Medical Center, 81 Irwon-ro Gangnam-gu, Seoul, 06351 Republic of Korea

**Keywords:** Cardiology, Medical research

## Abstract

Various efforts have been made to diagnose acute cardiovascular diseases (CVDs) early in patients. However, the sole option currently is symptom education. It may be possible for the patient to obtain an early 12-lead electrocardiogram (ECG) before the first medical contact (FMC), which could decrease the physical contact between patients and medical staff. Thus, we aimed to verify whether laypersons can obtain a 12-lead ECG in an off-site setting for clinical treatment and diagnosis using a patch-type wireless 12-lead ECG (PWECG). Participants who were ≥ 19 years old and under outpatient cardiology treatment were enrolled in this simulation-based one-arm interventional study. We confirmed that participants, regardless of age and education level, can use the PWECG on their own. The median age of the participants was 59 years (interquartile range [IQR] = 56–62 years), and the median duration to obtain a 12-lead ECG result was 179 s (IQR = 148–221 s). With appropriate education and guidance, it is possible for a layperson to obtain a 12-lead ECG, minimizing the contact with a healthcare provider. These results can be used subsequently for treatment.

## Introduction

It is critical to obtain a 12-lead electrocardiogram (ECG) as early as possible in patients with acute cardiovascular disease (CVD)^1–4^. CVD reportedly claimed an estimated 17.8 million lives worldwide in 2017 and is currently the most prevalent non-communicable disease^5^. Twelve-lead ECGs are the most frequently used primary diagnostic tools for CVD; they are non-invasive and require only a short examination time^6^. Early obtainment of a 12-lead ECG in patients exhibiting relevant symptoms reportedly reduces the in-hospital treatment time and improves the 30-day mortality^7–13^.

Emergency medical service (EMS) delays can be reduced by training healthcare providers to detect acute CVD in patients and implementing standardized treatment protocols^2,14^. However, the problem of patient delays persists as there is no solution other than public awareness campaigns; thus, it remains unaddressed^15^. In addition to the psychological considerations, barriers associated with unusual symptoms or misunderstandings delay patients’ decisions to visit a hospital or contact emergency services for assistance^16^.

Despite a decrease in hospitalization rate for acute myocardial infarction (AMI) during the coronavirus disease (COVID-19) pandemic^17,18^, the overall treatment time for AMI has reportedly increased^19,20^, with a greater delay in the time to first medical contact (FMC)^21^. One potential explanation for this phenomenon is a combination of strain on healthcare resources^22,23^ and patient-related factors such as job loss-related financial concerns about medical expenses^17^ and reluctance to visit hospitals due to the risk of contracting COVID-19^24,25^.

Considering that early obtainment of a 12-lead ECG improves outcomes^1–4,7–12^, if patients are able to independently measure and manage the 12-lead EGC, even before FMC, —can be a solution for those with delays and constraints on medical resources can be avoid.

Although watch- or ring-type wearable technology devices have been created for ECG measurements by laypersons, they are not ideal for diagnosing CVD^26^. Several studies have made it possible for the general public to use 12-lead ECGs. However, one study used a calculated 12-lead ECG rather than a direct measurement^27^, and another study focused on the development process rather than on actual patients^28^. Another study utilized the commonly worn Apple Watch; however, this method has the limitation of not being able to measure all 12 leads simultaneously, requiring each lead to be measured individually^29^. Currently, there is no technology, whose performance has been validated, that enables laypersons to independently measure 12-lead ECGs^30^.

We aimed to develop a patch-type wireless 12-lead ECG (PWECG) that allows a layperson to acquire and monitor a 12-lead ECG in an off-site environment for early diagnosis and treatment of AMI.

## Methods

### Study design and setting

The experimental study, with one intervention arm, was conducted at a university hospital in Seoul to verify the hypothesis that a layperson can attach a PWECG and obtain results within ten minutes. We prepared a separate room, with just a bed, outside the main hospital building to simulate a scenario of the participant using the device at home. To protect patient privacy, the study was conducted at a secure area and female researchers were designated to supervise female participants. This study was approved by the Institutional Review Board (IRB) of the Samsung Medical Center (IRB no. 2019-04-059) and performed in accordance with the principles of the Declaration of Helsinki. All participants voluntarily participated in the study and provided written informed consent before enrollment. The entire study process was performed according to the protocol approved by the Samsung Medical Center IRB.

### Study population and sample size

We established an alternative hypothesis that the time required for a layperson to acquire a 12-lead ECG differed from the reference value. The reference value of 600 s was set because the European Resuscitation Council (ERC) recommends obtaining a 12-lead ECG within 10 min from FMC^14^. We used a one-sample t-test to determine the sample size following a six-person pilot test. The primary outcome was the average time (in seconds) from the starting point of taking off the shirt to acquiring the 12-lead ECG. The six-person pilot study determined that this mean time was 407.83 s with a standard deviation of 208.968 s. Based on this, the total sample size was calculated as 19, assuming a significance level of 5%, power of 90%, and failure rate of 20%. The determined population was allocated by age and sex based on the number of patients with AMI visiting the emergency room (ER)in Korea, as released by the Korea National Statistics Office^31^.

Patients who had received healthcare provider education (doctors, nurses, emergency medical technicians, etc.) were excluded from the study to ensure that the self-attachment and acquisition of 12-lead ECG was performed by a layperson. In addition, those who had recently undergone thoracic surgery, pregnant women, and patients with an implantable cardioverter defibrillator were excluded from the study.

The inclusion criteria were adults aged ≥ 19 years who were undergoing outpatient cardiology treatment and voluntarily agreed to participate in the study. A recruitment poster containing the purpose and detailed information of the study was posted to the outpatient clinic notice board.

### Materials

**Figure 1:**
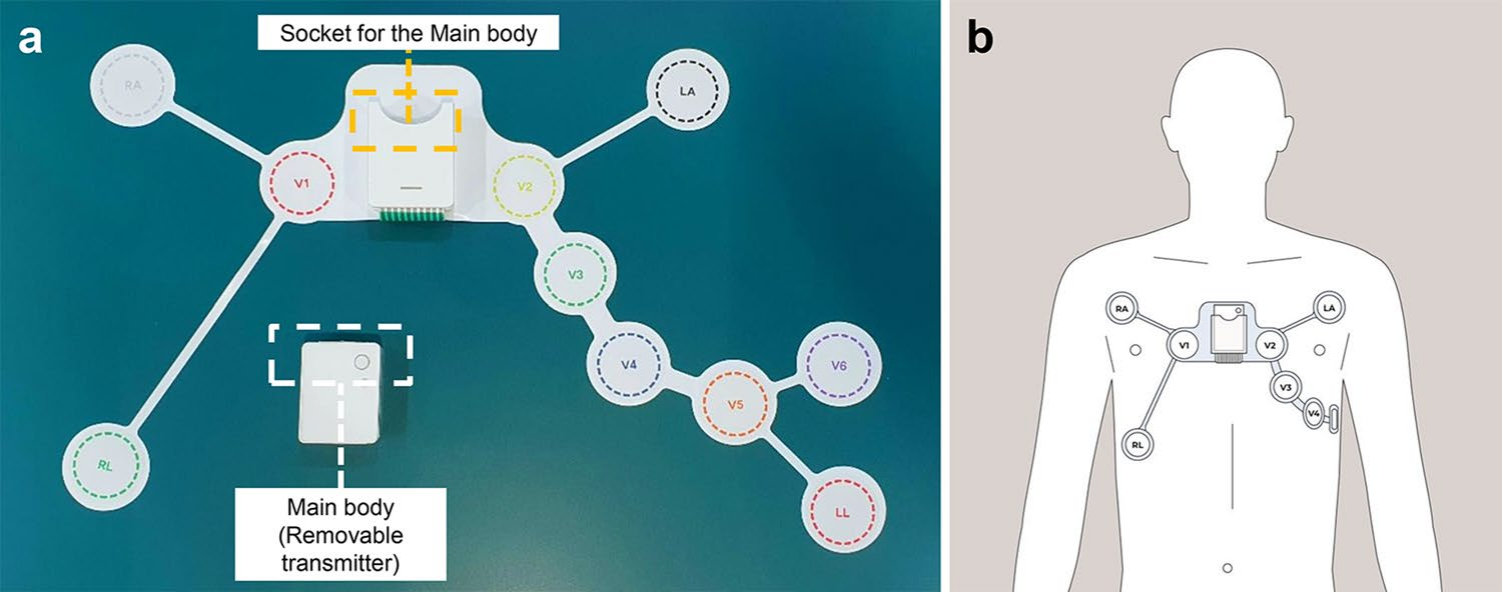
(**a**) Device used for the study (Healthrian, Yuseong-gu, Daejeon, Republic of Korea). The device comprises two parts, a flexible single patch with a socket and a main body. A 12-lead ECG will be produced by the six precordial and four limb leads that are attached to the chest surface. The recorded ECG was saved to the tablet and main body. (**b**) Appropriate attachment of the flexible patch to the patient’s chest and assembly of the main body in the socket.

The PWECG intervention device used in this study was the WearECG12 (Healthrian), which has been approved by the Korean Ministry of Food and Drug Administration as a Holter ECG (Fig. 1). It comprises a main body and patch-type electrode. The main body measures 46 mm * 35.6 mm * 16 mm and weighs 30 g; the patch measures 241.19 mm * 375.5 mm and weighs 35 g. The device collects a 12-lead ECG and has filters such as a notch, low-pass, and high-pass. The data were stored for approximately 60 h at a sampling frequency of 250 sps. The main body was assembled in a socket on the patch. To perform a 12-lead ECG examination and continuous monitoring, the tablet and the main body are wirelessly connected via Bluetooth. The tablet used in this study was the Samsung Galaxy Tab A 8.0 (SM-T380), Wi-Fi model.

### Study protocols

All the participants included in this study provided written consent, stating their voluntary participation. Basic information that could affect the study results were collected, such as sex, age, past medical history (hypertension [HTN], diabetes mellitus [DM], AMI, and stroke), educational background, and number of years of smartphone use.

Each participant was pre-trained by an emergency medical physician for a minimum of 20 min. First, the components and detailed procedures of the PWECG were explained to the participants with written guidelines. Second, the appropriated method of ECG attachment was repeatedly practiced by the participants on a training mannequin. The exact placement of the main patch and V1 and V2 electrodes to the center of the chest was guided by medical personnel. The practice was conducted in the same order as that of the study: accurate patch placement, Bluetooth pairing of the main body and tablet, and acquisition of a 12-lead ECG. The tablets used during the practice were the same as those used during the study.

All participants began the study while wearing a similar shirt. Without the aid of written instructions or the researcher’s guidance, participants completed the entire test process of patch attachment and 12-lead ECG obtainment. The researchers measured the time required for each step using a stopwatch. The applications used in this study had built in automatic 12-lead ECG transfer functions; however, this function was switched off to verify a laypersons’ ability to perform it.

**Figure 2:**
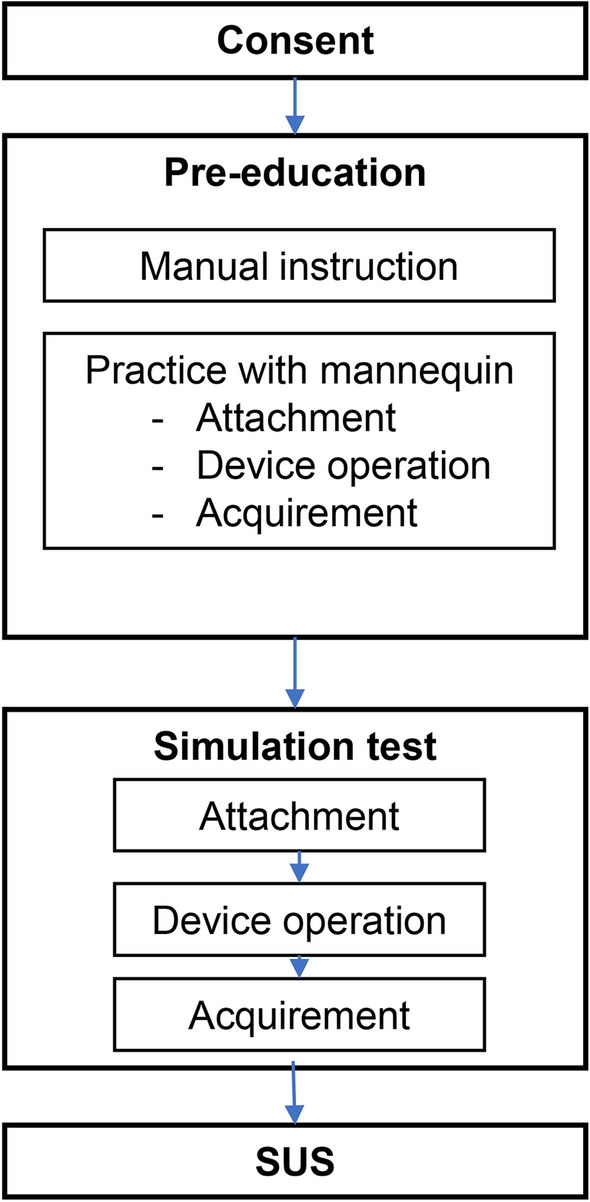
Study process. Pre-education included visual instruction and actual practice. Mannequins were used during the entire process.

After all the procedures were completed, participants filled out a system usability scale (SUS) survey of the PWECG device (Fig. 2).

### Outcome measure

**Figure 3:**
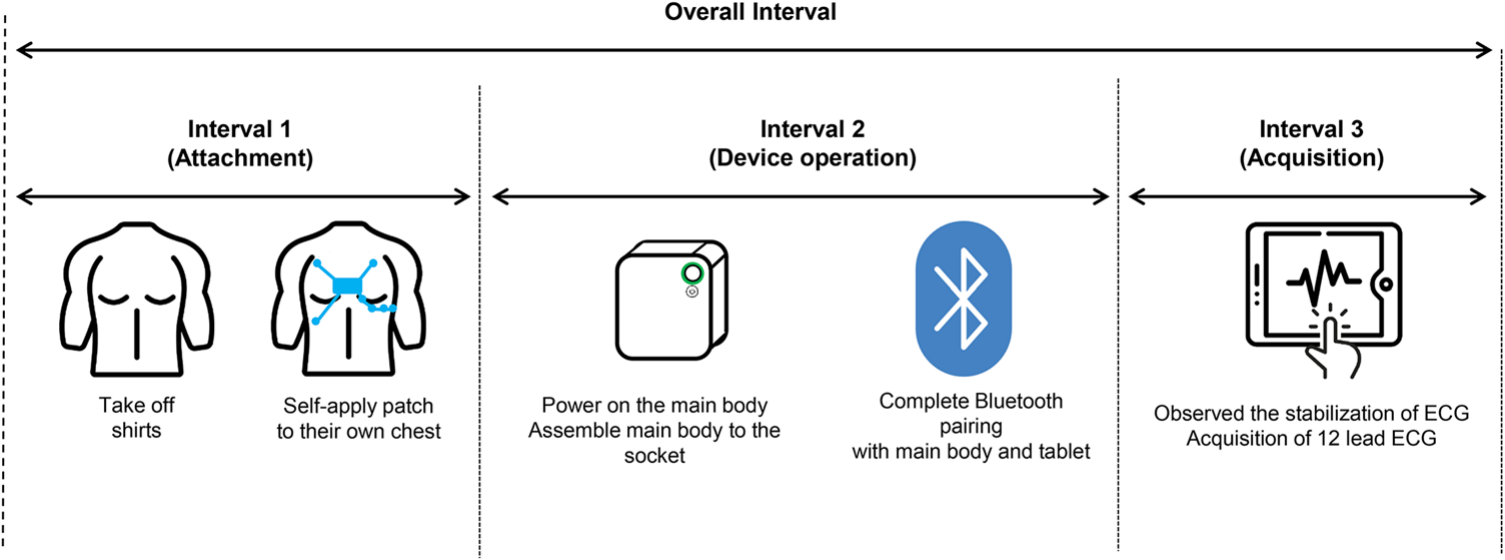
Overall study process comprised three intervals. Attachment step: self-application of the patch to the appropriate chest location. Device operation step: connection of the device to the tablet via Bluetooth. Acquisition step: obtainment of a stable 12-lead ECG.

The primary outcome was the time interval (in seconds) from taking off the patient’s shirt to acquisition of a 12-lead ECG result on the tablet. The overall time interval was divided into three stages (patch attachment, device operation, and acquisition of the 12- lead ECG results). If there was a failure or delay in any stage, the problems and reasons were recorded by the researcher (Fig. 3). The entire interval was recorded by the researcher using a stopwatch.

1. Interval 1 (Attachment): From shirt removal to the completion of patch self-attachment (including modifying the patch location). In this step, we aimed to evaluate the ability of participants to use a novel medical device in accordance with their training.
2. Interval 2 (Operation): From switching on the power of the main body to completion of Bluetooth pairing with the tablet. This represents a fundamental skill in creating and communicating personal health records using mobile devices.
3. Interval 3 (Acquisition): From observation of ECG stabilization to acquisition of the 12-lead ECG. In this step, participants were required to select a single, stable ECG record from among various options. They had the option of reattempting to capture a stable ECG, if desired.

The secondary outcome was the SUS, which represented the laypersons’ satisfaction with the device usability. The SUS comprises ten questions on a 5-point Likert scale, with one indicating strongly disagree and five indicating strongly agree. The total SUS result was calculated by subtracting 1 from the score for each odd item and subtracting 5 for each even number item; subsequently, the results were added and multiplied^32^. The final calculated SUS was interpreted based on a previous study^33^: > 90.9 points was “best imaginable”; 85.5–90.9 was “excellent”; 71.4–85.4 was “good”; 50.9–71.3 was “OK”; 35.7–50.8 was “poor”; 20.3–35.6 was “awful”; and 12.5–35.5 was “worst imaginable”.

### Data analysis

We used the Wilcoxon test to analyze single-sample linear regression and observe the relationship between two continuous variables. The Kruskal–Wallis test was used for subgroup analysis. P values less than 0.05 were considered statistically significant. All data processing and statistical analyses were conducted using R (version 3.6.3; R Foundation for Statistical Computing, Vienna, Austria).

## Results

### Participant characteristics

Eighteen of the 19 participants succeeded in acquiring the 12-lead ECG results. In the single, unsuccessful case, the Bluetooth connection could not be established at interval 2 because of a device-related problem on the tablet. The researcher intervened and connected the Bluetooth; the overall interval including the researcher's intervention was 381 s.

The study was initially planned to recruit five women; however, only two were finally included because the rest could not be recruited (Table 1). The median age of the participants was 59 years (IQR = 56–62 years), and 61% had a university education or higher. All participants had been using smartphones at the time of the study, with a median duration of 10 years (IQR = 7–10 years). The total number of participants with more than one medical history was 61%, with the most common disease being MI (50%).

**Table 1:** Basic characteristics of the participants.

Characteristic	Number
Median age (IQR) (years)	59 (56–62)
Age-group, n (%)	
20–39 years	3 (16.7%)
40–49 years	1 (5.6%)
50–59 years	6 (33.3%)
60–69 years	8 (44.4%)
Gender, n (%)	
Female	2 (11.1%)
Male	16 (88.9%)
Education, n (%)	
Middle-school graduates	2 (11.1%)
High-school graduates	5 (27.8%)
University graduates	11 (61.1%)
Years of smartphone use, median (IQR)	10 (7–10)
Past medical history, n (%)	
None	7 (38.9%)
Hypertension^a^	4 (22.2%)
Diabetes mellitus^b^	3 (16.7%)
Myocardial infarction^c^	9 (50.0%)
Stroke^d^	1 (5.6%)

^a^Participants who only had hypertension were 2.

^b^Participants who only had diabetes mellitus were 1.

^c^Participants who only had a myocardial infarction were 6.

^d^Participants who only had a stroke were 1.

### Time interval outcome

**Figure 4:**
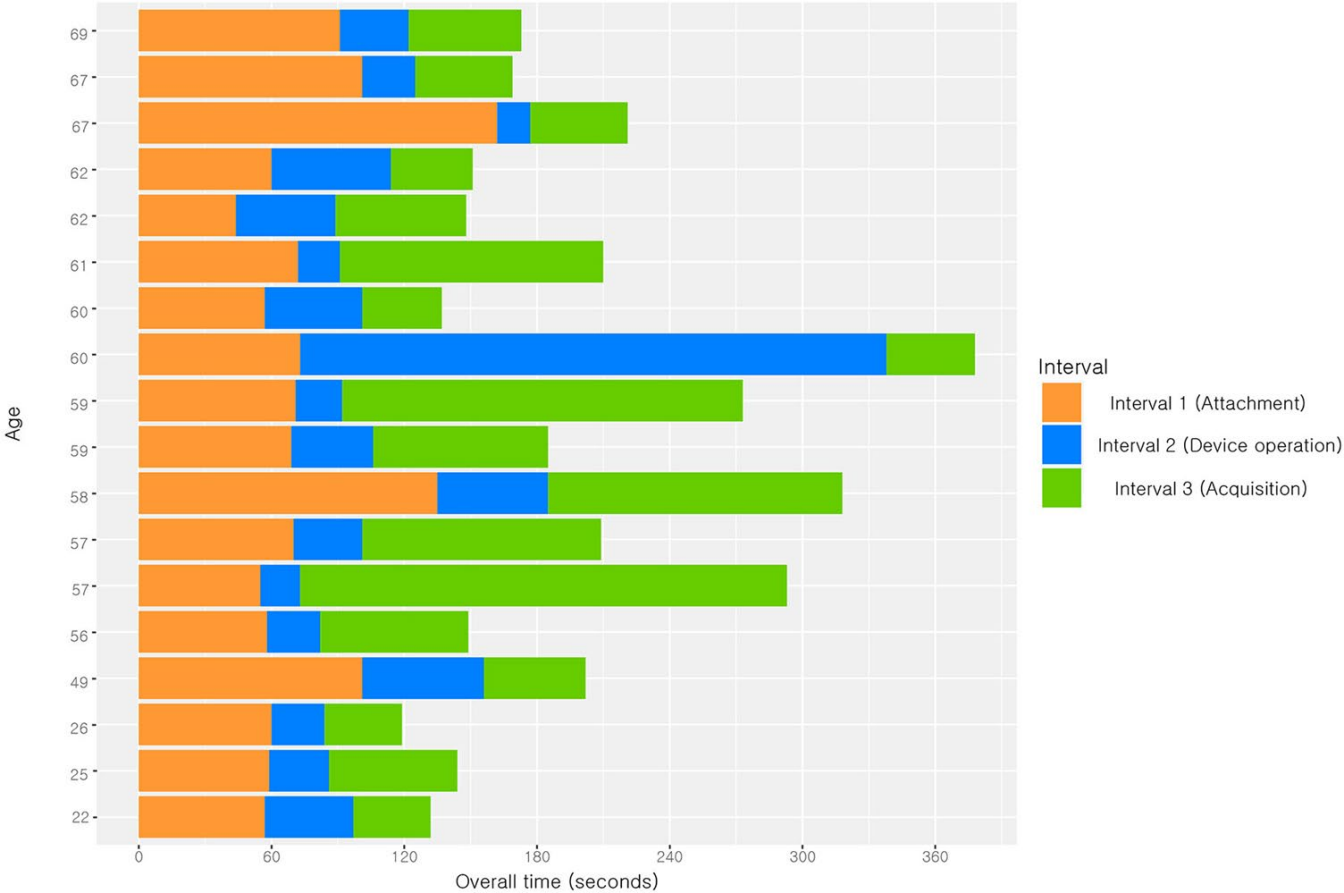
Bar chart of all participants’ age and interval outcome.

The median overall interval time among the 18 participants was 179 s (IQR = 148–221 s) and the maximum was 378 s (Table 2). The median time of interval 1 was 70 s (IQR = 58–91 s); the minimum was 44 s and the maximum was 162 s. The median time of interval 2 was 31 s (IQR = 24–45 s); the minimum was 15 s and the maximum was 265 s. The median time of interval 3 was 55 s (IQR = 40–108 s); the minimum was 40 s and the maximum was 220 s. On average, the most time-consuming stage was interval 1, whereas the most variable stage was interval 3. Interval 2 was the fastest on average with the smallest variation, except for one outlier (265 s). There was a tendency for increased time interval with age; however, this was not statistically significant. The ages of all the participants and the time required for each phase are shown in Fig. 4.

**Table 2:** Time taken for each interval of patch-type wireless ECG device use.

Interval	Interpretation of interval	Time (seconds)
		Median (IQR)
1 (Attachment)	From shirt removal to completion of patch self-attachment	70 s (58–91 s)
2 (Device operation)	From switching on the power on the main body to completion of Bluetooth pairing	31 s (24–45 s)
3 (ECG acquisition)	From observing ECG stabilization to acquiring a 12-lead ECG	55 s (40–108 s)
Overall		179 s (148–221 s)

*ECG* electrocardiogram.

The analysis of the overall interval time according to age groups, > 60 years and < 60 years, showed no statistically significant difference (p = 0.96). Based on the level of education, the university group (202 s) took 29 s longer than the middle and high school graduation group (173 s); the result was not statistically significant (p = 0.53). Sub-analysis using the Kruskal–Wallis test, based on smartphone usage (< 10 years, 10 years, > 10 years) was also not found to be statistically significant (p = 0.53) (Table 3).

**Table 3:** Comparison of the overall time according to age, education, and smartphone use.

Category		N	Time (seconds)	p-value
			Median (IQR)	
Age	Up to 59 years	10	194 s (145–257 s)	0.96^a^
	Above 60 years	8	171 s (150–213 s)	
Education level	University	11	202 s (150–242 s)	0.53^a^
	Middle & high school	7	173 s (143–203 s)	
Smartphone use	> 10 years	8	156 s (136–234 s)	0.53^b^
	10 years	6	179 s (155–203 s)	
	< 10 years	4	206 s (189–231 s)	

^a^p-value was calculated using Wilcoxon sign rank sum test.

^b^p-value was calculated using Kruskal–Wallis test.

Successfully Bluetooth pairing on the first try was achieved in nearly all participants; only one participant repeated the process because he felt that he had failed. His recorded time was 378 s, which was the longest; interval 2 lasted 265 s.

### SUS results

The mean SUS survey result was 76.11 (Table 4), which indicates that the participants felt that the PWECG usability was good and is acceptable to the user. The lowest SUS value was 57.5, (“OK” usability), and the highest was 95 (“excellent” usability). Subgroup analysis of the association between SUS score and overall time showed that if the SUS score increased by 1 point, the overall time tended to decrease by 2 s; however, this result was not statistically significant (p = 0.15). Furthermore, the Kruskal–Wallis analysis of the overall time and SUS scores grouped in quarters was also not found to be statistically significant (p = 0.08).

**Table 4:** System usability scale results adapted for evaluation of participant satisfaction with the patch-type wireless device.

Question	Mean (SD)
1. I think that I would like to use the ECG device frequently	4.16 (0.61)
2. I found the ECG device unnecessarily complex	2.22 (1.21)
3. I thought the ECG device was easy to use	4.22 (0.73)
4. I think that I would need the support of a technical person to be able to use the ECG device	2.16 (1.09)
5. I found that the various functions in the ECG device were well-integrated	3.94 (0.63)
6. I thought there was too much inconsistency in the ECG device	2.00 (0.84)
7. I would imagine that most people would learn to use the ECG device very quickly	4.05 (0.72)
8. I found the ECG device very cumbersome to use	1.89 (0.83)
9. I felt very confident using the ECG device	4.44 (0.70)
10. I need to learn a lot of things before I could get going with the ECG device	2.11 (0.83)
Total score	76.11 (11.76)

*SD* standard deviation, *ECG* electrocardiogram.

## Discussion

This study confirmed that laypersons can perform 12-lead ECG examinations by themselves and obtain results within ten minutes through a mobile device. This study also used a new device designed for easy use by integrating the chest and limb leads into a single patch, rather than a device with conventional complex structures. During this study, the longest it took to complete the process was 378 s, thus confirming that even laypersons can apply and obtain a 12-lead ECG, with relative ease within ten minutes. This indicates that the 12-lead ECG area can be expanded from the medical line, and diagnostic and treatment data can be obtained by the patients themselves (Supplementary Fig. 1).

Due to the coronavirus pandemic, telemedicine, which reduces the exposure to patient risk and limits place^34–36^, is emerging as an important source of obtaining and sharing high-quality clinical information outside the hospitals for smooth clinical care. New technologies and services require progress in the areas of great clinical importance and prioritizing areas where patients can obtain 12-lead ECGs for the early diagnosis of potential AMI and arrhythmia. Patients obtaining a 12-lead ECG by themselves will reduce exposure risk if symptoms occur during their daily life or self-quarantine period.

In this study, the repeated Bluetooth pairing despite successful pairing on the first attempt by one patient may be presumed to be the participant’s inability to notice the connection. Considering that early stage of CVD is a rare and stressful situation, a 12-lead ECG must be properly accessible and easy to use. A previous study investigated the relationship between basic life support training time and the proportion of correct responses; the accuracy decreased after 6 months regardless of the training time^37^. Delays in steps related to device operation may resolve once users become accustomed to them; however, it also suggests that detailed user guides need to be provided for new devices and services, with accessibility to re-learn whenever necessary. Step-by-step audio guides, such as those used in an automated external defibrillator, may be considered. Furthermore, laypersons would require repeated training to maintain their proficiency.

This study has some limitations. First, because the calculated sample size was relatively small, it may be difficult to reflect the potential of the entire AMI patient population. This study was a simulated setting of the occurrence of AMI and not a real emergency situation. Second, there may be a selection bias because more than half of the participants were highly educated and non-smartphone users were not included in the study. However, as smartphone use becomes more common, task performance does not differ significantly according to educational level. This was consistent with our statistically insignificant findings regarding smartphone use and task time. Third, only two female participants were included. Our target female population was five; however, only two could be recruited. Considering that 31% of patients with AMI in Korea who visited the ER in 2017 were women, our study’s 11% female participation should be construed as restrictive. Fourth, although the difference in intervals was confirmed, the fundamental reason could not be investigated. In the future, laypersons’ thoughts and behavioral problems in the process of using new devices need to be analyzed through observations and in-depth interviews. Fifth, in this study, since the patient measured the feasibility of PWECG, a quality comparison of the collected ECG could not be conducted. In order to be able to actually utilize the 12-lead ECG obtained by the patient, it is necessary to compare it with a standard ECG to ensure that there are no quality problems.

In this study, we confirmed that it is feasible for laypersons to obtain 12-lead ECGs on their own using a PWECG, regardless of their age and education level.

## Supplementary Information

Supplementary Figure 1.

## Data Availability

The datasets generated and analyzed during the current study are not publicly available because of the small sample size and de-identification issues. However, they can be made available upon reasonable request to the corresponding author.

## Abbreviations

AMI: Acute myocardial infarction
COVID-19: Corona virus disease
CVD: Cardiovascular disease
DM: Diabetes mellitus
ECG: Electrocardiogram
EMS: Emergency medical service
ER: Emergency room
ERC: European resuscitation council
FMC: First medical contact
HTN: Hypertension
IRB: Institutional review board
MI: Myocardial infarction
PWECG: Patchy type wireless 12-lead electrocardiogram
SUS: System usability scale
