# Supplementary material for: Feasibility of patch-type wireless 12-lead electrocardiogram in laypersons
Source: Sci Rep. 2023 Mar 10;13:4044. doi: 10.1038/s41598-023-31309-0 (PMC10004446; doi:10.1038/s41598-023-31309-0)

**Supplementary Figure 1.** Example of a 12-lead electrocardiogram obtained from this study. Each of the images (a, b, c, d, and e) is a 12-lead electrocardiogram result performed by different participants themselves.


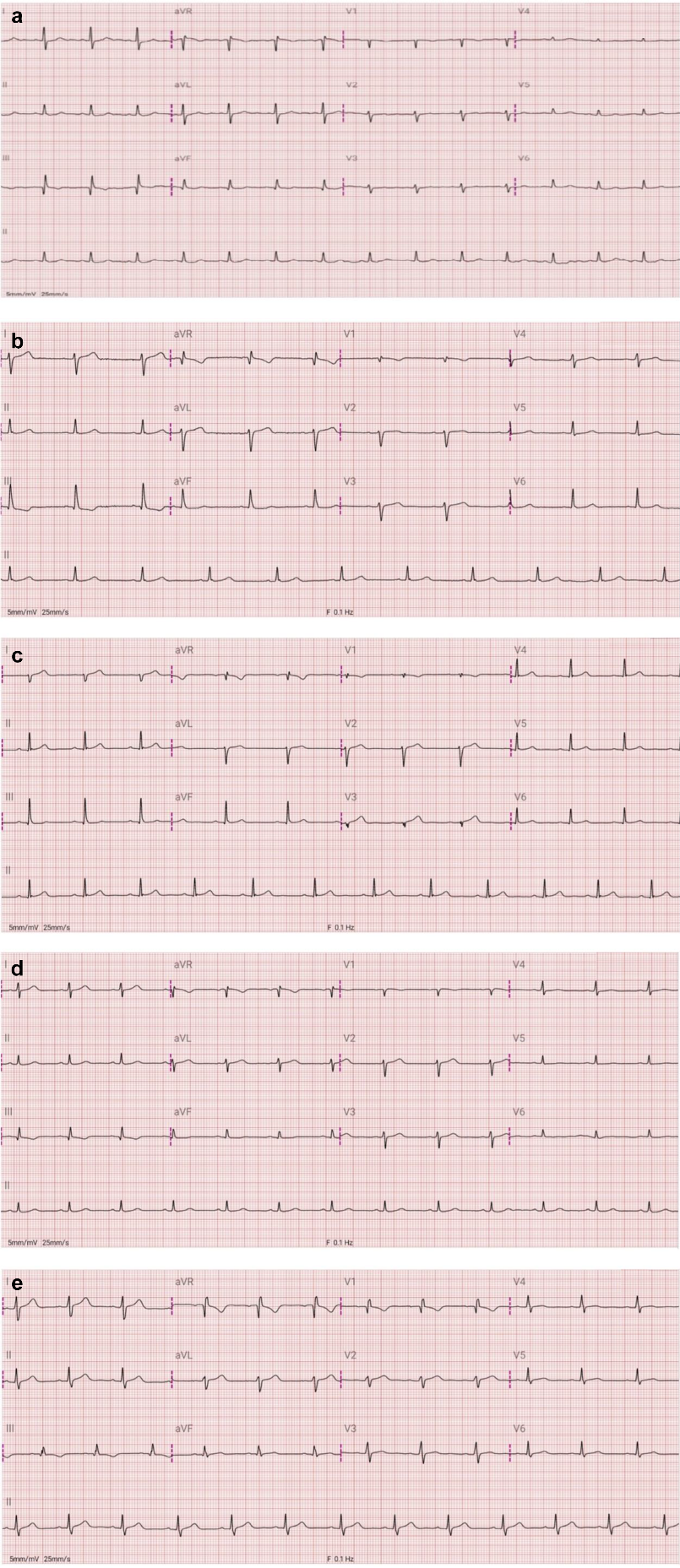

Supplement: Supplementary file 1 — Supplementary Figure 1. [file 41598_2023_31309_MOESM1_ESM.docx]
